# The Impact of Progesterone Receptor Status on Survival Outcomes in Metastatic Breast Cancer Patients Treated with First-Line CDK4/6 Inhibitors

**DOI:** 10.3390/cancers17040693

**Published:** 2025-02-18

**Authors:** Murad Guliyev, Ali Kaan Güren, Emre Özge, Rumeysa Çolak, Nargiz Majidova, Gülin Alkan Şen, Shamkhal Safarov, Murat Günaltılı, Mehmet Cem Fidan, İlkay Gültürk, Mesut Yılmaz, İbrahim Vedat Bayoğlu, Nebi Serkan Demirci, Özkan Alan

**Affiliations:** 1 Division of Medical Oncology, Department of Internal Medicine, Cerrahpaşa Faculty of Medicine, Istanbul University-Cerrahpaşa, Istanbul 34098, Türkiye; gulinalkan@msn.com (G.A.Ş.); shamxalnn@gmail.com (S.S.); muratgunaltili@hotmail.com (M.G.); mcemfidan@hotmail.com (M.C.F.); drserkannebi@yahoo.com (N.S.D.); ozkan.alan@hotmail.com (Ö.A.); 2 Division of Medical Oncology, Department of Internal Medicine, School of Medicine, Marmara University, Istanbul 34899, Türkiye; alikaanguren@gmail.com (A.K.G.); nergiz.mecidova1991@gmail.com (N.M.); dr.vebay@gmail.com (İ.V.B.); 3 Department of Medical Oncology, University of Health Science, Istanbul Training and Research Hospital, Istanbul 34098, Türkiye; emre_ozge89@hotmail.com (E.Ö.); gulturkilkay@gmail.com (İ.G.); 4 Department of Medical Oncology, University of Health Science, Bakirkoy Dr. Sadi Konuk Training and Research Hospital, Istanbul 34147, Türkiye; colak.rmys@gmail.com (R.Ç.); mesutyilmaz12@yahoo.com (M.Y.)

**Keywords:** breast cancer, progesterone receptor, CDK4/6, prognosis, survival

## Abstract

Background/Objectives: CDK4/6 inhibitors have changed the landscape of hormone receptor (HR)-positive, human epidermal growth factor receptor 2 (HER2)-negative metastatic breast cancer (BC) management. It is essential to identify predictive and prognostic factors for the efficacy of CDK4/6 inhibitors. We aimed to investigate the differences in characteristics and outcomes of patients receiving first-line CDK4/6 inhibitors according to PgR status. Methods: This multicenter retrospective study included 351 patients treated with first-line CDK 4/6 inhibitors for HR-positive/HER2-negative metastatic BC. Patients were categorized based on their PgR expression levels, including the PgR-low (<20%) and PgR-high (≥20%) groups, and baseline characteristics, treatment responses, and survival outcomes were analyzed. Results: The median age was 57 years (range: 26–85). A total of 99 patients were premenopausal, and 252 patients were postmenopausal. There were 115 (32.8%) patients in the PgR-low group, while 236 (67.2%) were in the PgR-high group. The majority of patients (56.7%) presented with de novo metastatic disease. Visceral metastases presented in 44.2% of patients. Low PgR expression was significantly associated with lower estrogen receptor levels (*p* = 0.031), elevated Ki-67 levels (*p* = 0.002), a higher incidence of visceral metastases (*p* = 0.035), and recurrent disease (*p* = 0.019). In the multivariate analysis, low PgR expression was a significant independent predictor of worse progression-free survival (PFS) and overall survival (OS). Conclusions: We demonstrated that low PgR expression is independently and significantly correlated with shorter PFS and OS. These findings support low PgR expression as a valuable prognostic biomarker in metastatic BC patients treated with first-line CDK4/6 inhibitors.

## Simple Summary

Over the past eight years, the addition of the cyclin-dependent kinase (CDK) 4/6 inhibitors in the management of patients with metastatic hormone receptor-positive breast cancer has dramatically improved clinical outcomes. Nevertheless, early progression may occur in a notable percentage of patients, and biomarkers that can predict treatment failure have not yet been precisely defined. Progesterone receptor (PgR) status is recognized as a significant biomarker for predicting prognosis in hormone-positive breast cancer; however, its impact on the efficacy of CDK 4/6 inhibitors remains a matter of debate. The aim of this study was to investigate the impact of PgR status on the prognosis of patients receiving first-line CDK4/6 inhibitors in combination with endocrine therapy. The expression level of PgR might be used as a cost-effective and accurate indicator for assessing the efficacy of CDK4/6 inhibitors.

## 1. Introduction

Breast cancer (BC) is the most common type of cancer in women worldwide and the second leading cause of cancer-related deaths [1]. BC is characterized by tumor heterogeneity, which encompasses various molecular subtypes and biological characteristics that result in distinct clinical behaviors and treatment responses. Hormone receptor (HR)-positive, human epidermal growth factor receptor 2 (HER2)-negative disease is the most common molecular subtype of BC, comprising approximately 60–70% of all cases [2].

Endocrine therapy (ET) remains its therapeutic backbone in patients with HR-positive/HER2-negative BC. Despite developments in early diagnostic and screening methods, as well as improved survival outcomes with adjuvant ET, a significant percentage of patients may develop de novo or recurrent metastatic disease [3,4]. The addition of cyclin-dependent kinase (CDK) 4/6 inhibitors (palbociclib, ribociclib, and abemaciclib) ushered in a new era in the treatment of metastatic HR-positive/HER2-negative BC [5]. Importantly, the findings from pivotal phase 3 studies indicate that the combination of ET with CDK4/6 inhibitors is the current standard approach in the first-line treatment of HR-positive/HER2-negative metastatic BC [6,7,8,9,10].

The combination of ET with CDK4/6 inhibitors generally results in a twofold increase in progression-free survival (PFS) compared to ET alone; however, a notable percentage of patients (15–25%) experience rapid progression regardless of the CDK4/6 inhibitors. Consequently, there is a necessity for the identification of biomarkers that can predict long-term benefits and early progression.

The progesterone receptor (PgR) belongs to the steroid hormone receptor family and is not exclusively an estrogen receptor-alpha (ERα)-induced gene target; it also functions as an ERα-associated protein that influences its activity [11]. PgR negativity is one of the significant risk factors for recurrence in ER-positive BC [12]. The expression of PgR has been identified as a significant prognostic indicator in assessing outcomes for patients with early-stage BC undergoing adjuvant ET [13,14,15,16]. Furthermore, lower PgR expression has also been observed to negatively impact survival in metastatic BC patients treated with ET [17,18].

The current literature presents limited and inconsistent results regarding the influence of PgR levels on survival outcomes in patients with HR-positive/HER2-negative metastatic BC treated with CDK 4/6 inhibitors [19,20,21,22,23]. In conclusion, additional studies are needed to further investigate this issue and determine the prognostic threshold value of PgR expression.

The aim of the present multicenter, observational study was to evaluate the impact of PgR expression level on the clinical outcomes of HR-positive/HER2-negative metastatic BC patients treated with first-line CDK4/6 inhibitors in combination with ET.

## 2. Materials and Methods

### 2.1. Study Design

This multicenter, retrospective study included 351 patients from four oncology clinics diagnosed with HR-positive/HER2-negative metastatic BC between January 2018 and January 2024 who received first-line ribociclib or palbociclib (abemaciclib is not covered by insurance in our country) in combination with ET. The criteria for inclusion were as follows: histopathological confirmation of BC; radiological evidence of metastatic disease; assessment of ER, PgR, and HER2 status through immunohistochemistry (IHC); patients who received at least two cycles of CDK4/6 inhibitors in combination with ET as first-line treatment; and radiological evaluation of treatment response. The exclusion criteria included patients lacking radiological assessment of treatment response, inadequate clinical documentation, and uncertain clinical outcomes.

### 2.2. Patient Population

We extracted clinicopathological data on patients from databases and medical records. Baseline characteristics, including age, sex, Eastern Cooperative Oncology Group (ECOG) performance status, menopausal status, histopathologic features of tumor, presence of de novo/recurrent metastatic disease, endocrine-sensitive/resistant status prior to the administration of CDK 4/6 inhibitors, anatomical location of metastases, and presence of visceral or non-visceral metastasis, and treatment-related toxicities, were collected along with survival data.

The status of endocrine resistance prior to the initiation of CDK 4/6 inhibitors was defined in accordance with the 4th ESO–ESMO International Consensus Guidelines for Advanced Breast Cancer [24]. We classified patients with de novo metastatic disease or patients who experienced recurrence more than 12 months post-adjuvant therapy as endocrine-sensitive. Patients exhibiting recurrence during adjuvant therapy or within the initial 12 months following the end of adjuvant therapy were classified as endocrine-resistant.

We conducted the assessments for ER, PgR, and HER2 status using available biopsy results from either the primary tumor or the metastatic lesion. For patients with recurrent metastatic disease, the biopsy result closest to the initiation of CDK4/6 inhibitor treatment was used. The assessment of ER, PgR, and HER2 status in patients was performed in accordance with the American Society of Clinical Oncology/College of American Pathologists (ASCO/CAP) guidelines [25,26]. Patients with a HER2 IHC score of 0 were categorized as HER2-negative, while those with a HER2 IHC score of +1, +2, and without gene amplification by in situ hybridization (ISH) were categorized as HER2-low. In accordance with the St. Gallen consensus recommendations [27], we determined the threshold for Ki-67 and PgR expression levels at 20%, and we categorized patients as Ki-67-low (<20%) and Ki-67-high (≥20%); PgR-low (<20%) and PgR-high (≥20%).

### 2.3. Efficacy and Safety Measures

The time from the start of treatment to disease progression or death from any cause, whichever occurred first, was defined as progression-free survival (PFS). Patients who did not experience progression were censored at their last follow-up visit. Overall survival (OS) was defined as the time from the start of systemic treatment until death from any cause or the last visit. Patients who were still alive were censored from the date of analysis. Treatment-related adverse events (TRAEs) were graded in accordance with the National Cancer Institute Common Terminology Criteria for Adverse Events (CTCAE), version 5.0 [28].

### 2.4. Ethical Considerations

This study was performed in accordance with the principles of the Declaration of Helsinki and was approved by the local ethics committee for clinical trials (date: 19 November 2024; number: E-83045809-604.01-1149120). According to the retrospective design of this study, the necessity for informed consent was waived.

### 2.5. Statistical Analysis

SPSS version 26 was used to perform the statistical analysis. First, normal distribution was tested for quantitative variables. Variables that exhibited a normal distribution were analyzed using a t-test between the two groups. If the variables did not meet this criterion, they were compared using the Mann–Whitney *U* non-parametric test. The baseline categorical characteristics of the patients were compared using either Fisher’s exact test or the chi-squared test. The Kaplan–Meier method was used to estimate survival curves, which were compared using the log-rank test. Multivariate analysis was carried out using the Cox proportional hazards model to assess the effect of prognostic factors on PFS and OS. Hazard ratios (HR) with 95% confidence intervals (CI) were also calculated. Statistical significance was defined as a *p*-value of less than 0.05.

## 3. Results

### 3.1. Baseline Characteristics of Patients

The study cohort consisted of 351 patients. All patients were female. The median age was 57 years (range: 26–85) at CDK 4/6 inhibitor initiation. The majority of patients were diagnosed with the histopathological subtype of invasive ductal carcinoma (70.7%). A total of 99 patients (28.2%) were premenopausal, and 252 patients (71.8%) were postmenopausal. All patients exhibited ER-high (>10%) expression. There were 115 (32.8%) patients with low PgR, while 236 (67.2%) patients presented with high PgR. The median ER expression value was significantly lower in the PgR-low group than in the PgR-high one (90% vs. 95%; *p* = 0.031). Of the patients, 37.5% had low Ki-67 (<20), while 62.5% had high Ki-67 (≥20). The PgR-low group had a significantly higher median Ki-67 expression level than the PgR-high group (30% vs. 20%; *p* = 0.002). The majority of patients (56.7%) presented with de novo metastatic disease. The incidence of de novo metastatic disease was significantly lower in the PgR-low group compared to the PgR-high group (47.8% vs. 61%; *p* = 0.019). The vast majority of patients (72.9%) were hormone-sensitive at the initiation of CDK 4/6 inhibitors. While the count of hormone-sensitive patients in the PgR-low group was numerically lower than that in the PgR-high group, the difference did not reach statistical significance (67% vs. 75.8%; *p* = 0.078). A significant proportion of the patients (44.2%) presented with visceral metastases. The incidence of visceral metastases was significantly higher in the PgR-low group compared to the PgR-high group (52.2% vs. 40.3%; *p* = 0.035). Furthermore, the incidence of liver (27% vs. 15.7%; *p* = 0.012) and brain metastases (7% vs. 2.1%; *p* = 0.024) was also significantly higher in the PgR-low group than in the PgR-high group. The baseline characteristics of the study population are summarized in Table 1.

### 3.2. Survival Outcomes

The median follow-up time was 25.2 (range: 1.5–82.6) months. The last follow-up date was November 1, 2024. By this date, 153 patients (43.6%) had experienced disease progression, and 73 patients (21.1%) had died. The median OS was not reached, and the median PFS was 35.1 months (95% CI: 27.9–42.3) in the entire study cohort.

**Figure 1:**
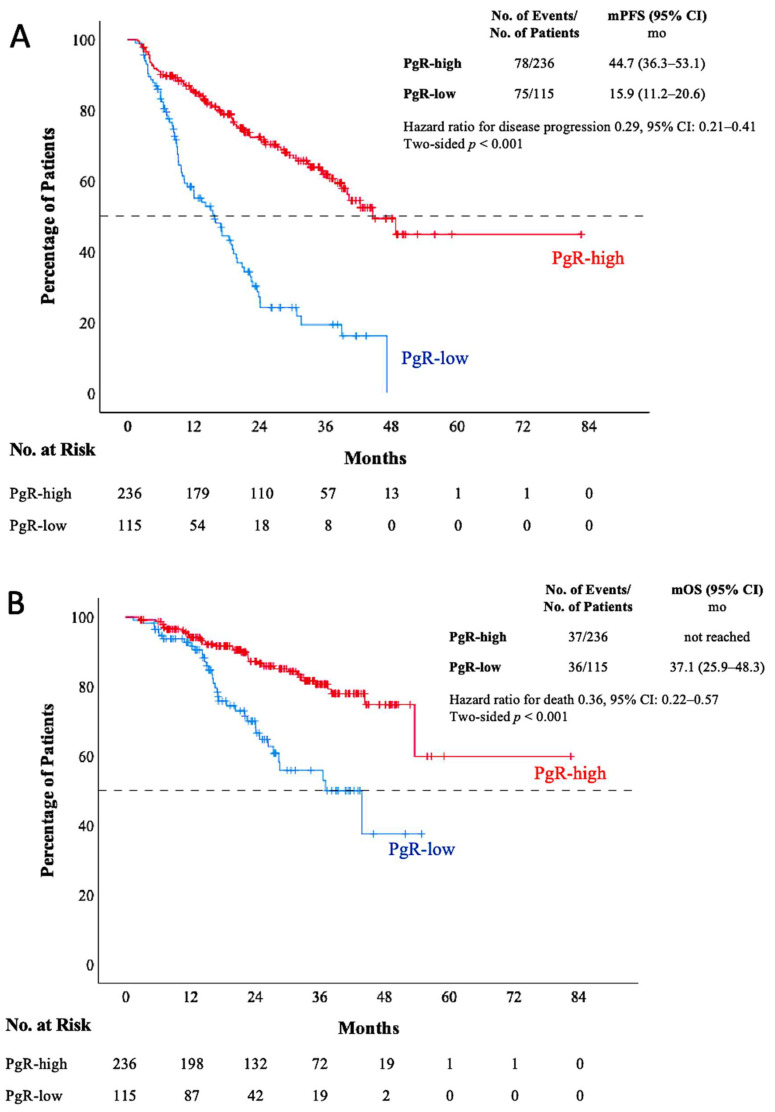
Kaplan–Meier curves of PFS (**A**) and OS (**B**) in patients with low PgR and high PgR. PgR: progesterone receptor; PFS: progression-free survival; OS: overall survival; mos: months; No.: number.

The median PFS was significantly lower in the PgR-low group compared to the PgR-high group (15.9 vs. 44.7 months; *p* < 0.001) (Figure 1A). In addition to low PgR expression levels, elevated BMI (≥30 vs. <30, *p* = 0.017), high tumor grade (3 vs. 1–2, *p* = 0.008), recurrent metastatic disease (*p* = 0.008), endocrine-resistant status (*p* < 0.001), presence of liver metastasis (*p* < 0.001), presence of non-only bone lesion (*p* < 0.001), and presence of brain metastasis (*p* < 0.001) were significantly correlated with poorer PFS in univariate analyses. Notably, low PgR expression levels (HR: 0.41, 95% CI: 0.28–0.61; *p* < 0.001), high tumor grade (HR: 1.55, 95% CI: 1.08–2.22; *p* = 0.019), endocrine-resistant disease (HR: 1.60, 95% CI: 1.10–2.31; *p* = 0.013), presence of liver metastasis (HR: 2.06, 95% CI: 1.34–3.17; *p* = 0.001), and presence of brain metastasis (HR: 2.28, 95% CI: 1.18–4.44; *p* = 0.015) were significant independent prognostic factors for decreased PFS in the multivariate analysis (Table 2).

The median OS was significantly lower in the PgR-low group compared to the PgR-high group (37.1 months vs. NR; *p* < 0.001) (Figure 1B). Additionally, premenopausal status (*p* = 0.039), high tumor grade (3 vs. 1–2, *p* = 0.015), presence of liver metastasis (*p* = 0.001), and presence of brain metastasis (*p* = 0.002) were significantly correlated with poorer OS in univariate analyses. Furthermore, low PgR expression levels (HR: 0.40, 95% CI: 0.24–0.68; *p* = 0.001), premenopausal status (HR: 2.35, 95% CI: 1.18–4.67; *p* = 0.015), high tumor grade (HR: 1.83, 95% CI: 1.10–3.02; *p* = 0.019), and the presence of liver metastasis (HR: 2.37, 95% CI: 1.33–4.21; *p* = 0.003) were significant independent prognostic factors for decreased OS in the multivariate analysis (Table 3).

### 3.3. Treatment Interventions

Of the patients, 245 (69.8%) were treated with ribociclib, while 106 (30.2%) were treated with palbociclib. The usage rates of ribociclib or palbociclib were similar between the PgR-low and -high groups (*p* = 0.056). Aromatase inhibitors (AIs) were the most frequently (76.4%) used ETs in combination with CDK 4/6 inhibitors. All patients with recurrent metastatic disease received adjuvant/neoadjuvant therapy and primary tumor resection at an early/locally advanced stage. Approximately only 3% of patients underwent surgery for the primary tumor while receiving CDK 4/6 inhibitor therapy.

### 3.4. Treatment-Related Adverse Events

Treatment-related adverse events (TRAEs) of any grade occurred in 40.7% of patients during CDK4/6 inhibitors in combination with ET (Table 4). Neutropenia was the most common TRAE, occurring in 34.8% of cases, followed by thrombocytopenia (2.8%), anemia (2.3%), and prolonged QTc (2%). The frequency of any-grade TRAEs did not significantly differ between the PgR-low and PgR-high groups (36.5% vs. 42.8%, *p* = 0.261). A total of 107 patients (30.5%) underwent dose modification of CDK 4/6 inhibitors due to TRAEs. Among these, the dose was reduced once in 87 patients (24.8%) and twice in 20 patients (5.7%). Dose modification was performed in 89 of 107 patients due to neutropenia. The incidence of dose-reducing TRAEs was numerically higher in the PgR-high group than in the PgR-low group, though this difference was not statistically significant (33.5% vs. 24.3%, *p* = 0.081). We observed no significant impact of either any-grade TRAEs or dose-reducing TRAEs on the median PFS (*p* = 0.829, *p* = 0.649, respectively).

## 4. Discussion

In the present study, we demonstrated that the lower PgR expression levels (<20%) were significantly associated with decreased OS and PFS in patients with HR-positive/HER2-negative metastatic BC treated with first-line CDK4/6 inhibitors. This association was independent of menopausal status, tumor grade, endocrine-resistant status, and the presence of liver or brain metastasis. Additionally, we revealed that patients with low PgR exhibited lower ER levels, elevated Ki-67 levels, and a higher incidence of recurrent disease and visceral metastases in comparison to the PgR-high patients.

The combination of CDK 4/6 inhibitors with ET is the standard-of-care treatment for patients with advanced-stage HR-positive/HER2-negative BC. These agents can also be administered as adjuvant therapy to patients with higher-risk early-stage disease [5]. Nevertheless, early progression may occur in a notable percentage of patients, and biomarkers that can predict treatment failure have not yet been precisely defined. Mutations in the estrogen receptor gene (ESR1), as well as alterations in the MAPK and PI3K pathways, are well known to lead to the development of resistance to CDK4/6 inhibitors [29]. Evaluating resistance mechanisms in every patient is frequently complicated by financial constraints and accessibility issues. The identification of cost-effective and easily accessible predictive and prognostic biomarkers for early progression with CDK 4/6 inhibitors is a crucial area of research.

PgR is a valuable prognostic biomarker in HR-positive BC. The absence of PgR expression reflected a nonfunctional ER pathway and less efficacy of ET [12]. Dunnwald et al. analyzed 155,175 female patients enrolled in the SEER program (1990–2001), finding that ER-positive/PgR-negative patients exhibited unfavorable characteristics and poorer survival rates compared to ER-positive/PgR-positive patients [30]. Dauphine et al. analyzed 826,599 female patients enrolled in the National Cancer Database (2004–2015), finding that single HR-positive BC subtypes (ER+/PgR- and ER-/PgR+) are more likely to have high-grade cancer, lymphovascular invasion, node-positive cancer, stage 4 cancer, a higher multigene assay score, and a worse prognosis than the ER+/PgR+ subtype [31].

The threshold value at which a PgR expression level significantly affects patients’ characteristics and survival rates is a matter of debate. ASCO/CAP guidelines [26] recommend defining ER-low positivity as if 1–10% of tumor cell nuclei are immunoreactive for ER and suggest that similar principles apply to PgR testing, which is used primarily for prognostic purposes in the setting of ER-positive BC. In the St. Gallen consensus, it was defined that the best cutoff for PgR positivity with characteristics of HR-positive breast cancer was at least 20% [27]. Kurozumi et al. [32] determined that PgR expression can be considered a significant prognostic indicator for assessing the long-term prognosis of ER-positive/HER2-negative BC, with an optimal cut-off value identified at 20%. The single-center prospective cohort study by Kwak et al. [33] assessed the impact of PgR expression levels on prognosis in young BC patients (<40 years). The findings indicated that patients with low (1–10%) and negative PgR (<1%) expression had a decreased OS in comparison to those with high PgR expression (>10%). Another study involving 863 luminal HER2-negative premenopausal and 1498 postmenopausal BC patients revealed that PgR expression levels represent a significant prognostic factor for premenopausal luminal/HER2-negative BC, with an optimal PgR cut-off point identified at 20% [34].

We established the threshold for PgR expression levels at 20% in accordance with the St. Gallen consensus [27] and corroborating studies [16,18,32,34,35,36] in the literature. In our study, significant differences were observed between the baseline characteristic features of PgR-low and PgR-high patients according to the 20% cut-off. We found that patients with low PgR had a higher incidence of visceral metastases than those with high PgR, consistent with previous studies [37,38,39]. We also revealed that low PgR expression was significantly associated with lower ER levels, elevated Ki-67 levels, and a higher incidence of recurrent disease compared to the PgR-high group.

There are limited studies in the literature evaluating the prognostic value of PgR expression levels on survival outcomes in metastatic BC patients treated with CDK4/6 inhibitors. In the exploratory analysis of the PALOMA-3 trial [40], evaluating predictors of prolonged benefit from palbociclib plus fulvestrant in women with endocrine-resistant BC, it was found that median PgR H-scores were higher among long-term responders regardless of the treatment group. A pooled analysis by the Food and Drug Administration (FDA) [19] indicated that all clinicopathological subgroups of patients with HR-positive, HER2-negative advanced BC derived benefits from CDK4/6 inhibitors, irrespective of PgR expression. Jia et al. [20] categorized 152 ER-high/HER2-negative BC patients receiving first-line CDK-4/6 inhibitors into three groups (PgR-low, -negative, and -high) by establishing a PgR level cut-off at 10%. The study revealed that PgR-negative patients had the shortest PFS and OS. There was no difference in survival between the PgR-low and -high groups. However, as the authors noted, the sample size of PgR-low patients in this study was limited. In another study, Shao et al. [21] retrospectively analyzed 81 patients treated with palbociclib and found that those with Ki67 < 30% and PgR ≥ 20% had a significantly longer PFS. A study by Berlanga et al. [22] including 210 metastatic BC patients treated with CDK4/6 inhibitors did not establish a cut-off for PgR expression levels. The patients were categorized into PgR-negative and PgR-positive groups, with the median PFS of the PgR-negative group being significantly shorter than that of the PgR-positive group (17.7 vs. 45.5 months, *p* = 0.02). Our findings demonstrated that low PgR expression (<20%) is an independent prognostic factor that negatively impacts both PFS and OS.

These real-world data results should be interpreted in light of several limitations. First, the retrospective acquisition of data from clinical databases can reveal potential selection biases and influencing factors that may affect the interpretation of the results. Secondly, this study focuses primarily on PgR expression without investigating other molecular characteristics (ESR1 mutation, PIK3CA and/or AKT signaling pathway alterations) that could potentially impact the prognosis or response to treatment. In another limitation, our study was unable to include patients who were receiving abemaciclib as it is not covered by insurance in our country. Incorporating these parameters in future studies would enhance the evaluation of the prognostic significance of low PgR expression in this patient population.

## 5. Conclusions

In conclusion, it is crucial to recognize the factors affecting the prognosis of HR-positive/HER2-negative metastatic BC patients treated with CDK4/6 inhibitors. Our findings support that low PgR expression may serve as a cost-effective and reliable biomarker for predicting prognosis in this patient population. Additional prospective studies are needed to verify the impact of PgR status on survival outcomes, particularly with the increasing use of CDK4/6 inhibitors.

## Figures and Tables

**Table 1:** Baseline characteristics of patients.

Variables		All Patients (*n* = 351)	PgR-Low (*n* = 115)	PgR-High (*n* = 236)	*p*-Value
Age (years)	Median (range)	57 (26–85)	57 (26–84)	57 (27–85)	0.753
	≥65, *n* (%)	118 (33.6)	37 (32.2)	81 (34.3)	0.689
Menopausal status, *n* (%)	Premenopausal	99 (28.2)	29 (25.2)	70 (29.7)	0.385
	Postmenopausal	252 (71.8)	86 (74.8)	166 (70.3)	
BMI (kg/m^2^)	Median (range)	27.6 (15.4–54.5)	27.6 (18.7–49.7)	27.3 (15.4–54.5)	0.442
Histopathology, *n* (%)	IDC	248 (70.7)	79 (68.7)	169 (71.6)	0.731
	ILC	38 (10.8)	12 (10.4)	26 (11)	
	Other	65 (18.5)	24 (20.9)	41 (17.4)	
Estrogen receptor level (%)	Median (range)	95 (15–100)	90 (30–100)	95 (15–100)	0.031
Ki 67 (%)	Median (range)	20 (1–90)	30 (1–90)	20 (1–80)	0.002
	≥20, n (%)	208 (62.5)	79 (71.8)	129 (57.8)	0.013
Tumor grade	Grade 1–2 Grade 3	187 (68.5) 86 (31.5)	59 (66.3) 30 (33.7)	128 (69.6) 56 (30.4)	0.585
HER-2 status, *n* (%)	Zero	287 (81.8)	93 (80.9)	194 (82.2)	0.761
	Low	64 (18.2)	22 (19.1)	42 (17.8)	
Metastatic disease, *n* (%)	De novo	199 (56.7)	55 (47.8)	144 (61)	0.019
	Recurrent	152 (43.3)	60 (52.2)	92 (39)	
Hormonal status, *n* (%)	Endocrine sensitive	256 (72.9)	77 (67)	179 (75.8)	0.078
	Endocrine resistance	95 (27.1)	38 (33)	57 (24.2)	
CDK 4/6 inhibitors, *n* (%)	Ribociclib	245 (69.8)	88 (76.5)	157 (66.5)	0.056
	Palbociclib	106 (30.2)	27 (23.5)	79 (33.5)	
Endocrine therapy, *n* (%)	Aromatase inhibitor	268 (76.4)	85 (73.9)	183 (77.5)	0.453
	Fulvestrant	83 (23.6)	30 (26.1)	53 (22.5)	
Bone lesion only, *n* (%)		104 (29.6)	28 (24.3)	76 (32.2)	0.130
Lymph node metastasis, *n* (%)		138 (39.3)	44 (38.3)	94 (39.8)	0.816
Visceral metastasis, *n* (%)		155 (44.2)	60 (52.2)	95 (40.3)	0.035
Lung metastasis, *n* (%)		90 (25.6)	30 (26.1)	60 (25.4)	0.894
Liver metastasis, *n* (%)		68 (19.4)	31 (27)	37 (15.7)	0.012
Brain metastasis, *n* (%)		13 (3.7)	8 (7)	5 (2.1)	0.024
Any grade TRAE, *n* (%)		143 (40.7)	42 (36.5)	101 (42.8)	0.261
Dose-reducing TRAE, *n* (%)		107 (30.5)	28 (24.3)	79 (33.5)	0.081

PgR: progesterone receptor, BMI: body mass index, IDC: invasive ductal carcinoma, ILC: invasive lobular carcinoma, HER: human epidermal growth factor receptor, CDK: cyclin-dependent kinase; TRAE: treatment-related adverse event.

**Table 2:** Univariate and multivariate Cox regression analyses for progression-free survival.

	Univariate			Multivariate		
Variable	HR	95% CI	*p*-Value	HR	95% CI	*p*-Value
Age (≥65 vs. <65 years)	0.88	0.62–1.24	0.456			
Menopausal status (pre vs. post)	1.01	0.71–1.44	0.970			
BMI (≥30 vs. <30 kg/m^2^)	0.64	0.45–0.92	0.017	0.71	0.46–1.08	0.109
Ki-67 (≥20 vs. <20%)	1.18	0.84–1.65	0.337			
Tumor grade (3 vs. 1–2)	1.62	1.14–2.32	0.008	1.55	1.08–2.22	0.019
HER-2 status (zero vs. low)	1.21	0.82–1.79	0.342			
Progesterone receptor (high vs. low)	0.29	0.21–0.41	<0.001	0.41	0.28–0.61	<0.001
Metastatic disease status (recurrent vs. de novo)	1.54	1.12–2.12	0.008	1.20	0.69–2.09	0.515
Hormonal status (endocrine resistant vs. sensitive)	1.96	1.41–2.72	<0.001	1.60	1.10–2.31	0.013
CDK 4/6 inhibitors (ribociclib vs. palbociclib)	1.26	0.91–1.76	0.170			
Lymph node metastasis, *n* (%)	1.26	0.91–1.74	0.156			
Liver metastasis (yes vs. no)	2.87	2.03–4.06	<0.001	2.06	1.34–3.17	0.001
Lung metastasis (yes vs. no)	1.20	0.84–1.75	0.312			
Bone lesion only (yes vs. no)	0.48	0.33–0.72	<0.001	0.66	0.42–1.05	0.077
Brain metastasis (yes vs. no)	3.54	1.95–6.40	<0.001	2.28	1.18–4.44	0.015
Any-grade TRAE (yes vs. no)	1.04	0.75–1.43	0.826			
Dose-reducing TRAE (yes vs. no)	0.92	0.65–1.31	0.649			

HR: hazard ratio; BMI: body mass index; HER: human epidermal growth factor receptor; CDK: cyclin-dependent kinase; TRAE: treatment-related adverse event.

**Table 3:** Univariate and multivariate Cox regression analyses for overall survival.

	Univariate			Multivariate		
Variable	HR	95% CI	*p*-Value	HR	95% CI	*p*-Value
Age (≥65 vs. <65 years)	1.50	0.94–2.41	0.089			
Menopausal status (pre vs. post)	1.88	1.03–3.42	0.039	2.35	1.18–4.67	0.015
BMI (≥30 vs. <30 kg/m^2^)	0.58	0.33–1.00	0.050			
Ki-67 (≥20 vs. <20%)	1.13	0.69–1.85	0.619			
Tumor grade (3 vs. 1–2)	1.86	1.13–3.08	0.015	1.83	1.10–3.02	0.019
HER-2 status (zero vs. low)	0.94	0.52–1.68	0.832			
Progesterone receptor (high vs. low)	0.36	0.22–0.57	<0.001	0.40	0.24–0.68	0.001
Metastatic disease status (recurrent vs. de novo)	0.99	0.62–1.57	0.961			
Hormonal status (endocrine resistant vs. sensitive)	1.27	0.77–2.08	0.346			
CDK 4/6 inhibitors (ribociclib vs. palbociclib)	1.19	0.74–1.91	0.479			
Lymph node metastasis, *n* (%)	0.91	0.57–1.48	0.713			
Liver metastasis (yes vs. no)	2.41	1.46–3.97	0.001	2.37	1.33–4.21	0.003
Lung metastasis (yes vs. no)	1.30	0.78–2.16	0.307			
Bone lesion only (yes vs. no)	0.69	0.40–1.17	0.169			
Brain metastasis (yes vs. no)	3.43	1.57–7.51	0.002	2.06	0.82–5.20	0.127
Any grade TRAE (yes vs. no)	1.11	0.70–1.76	0.656			
Dose-reducing TRAE (yes vs. no)	1.16	0.72–1.88	0.545			

HR: hazard ratio; BMI: body mass index; HER: human epidermal growth factor receptor; CDK: cyclin-dependent kinase; TRAE: treatment-related adverse event.

**Table 4:** TRAEs associated with the use of CDK4/6 inhibitors in combination with ET.

Adverse Event	Any Grade	Dose-Reducing TRAEs
	number of patients (percent)	
Neutropenia	122 (34.8%)	89 (25.4%)
Thrombocytopenia	10 (2.8%)	8 (2.3%)
Anemia	8 (2.3%)	4 (1.1%)
Prolonged QTc	7 (2%)	7 (2%)
Increased alanine aminotransferase	5 (1.4%)	3 (0.8%)
Increased aspartate aminotransferase	5 (1.4%)	3 (0.8%)
Creatinine increased	2 (0.6%)	2 (0.6%)
Diarrhea	2 (0.6%)	2 (0.6%)
Oral mucositis	1 (0.3%)	1 (0.3%)

TRAE: treatment-related adverse event; CDK: cyclin-dependent kinase; QTc: QT corrected.

## Data Availability

The data presented in this study are available on request from the corresponding author.
